# Novel phenotype with prominent cerebellar oculomotor dysfunction in spastic paraplegia type 39

**DOI:** 10.1007/s00415-022-11313-6

**Published:** 2022-08-10

**Authors:** Sebastian Viertauer, Ingo Kurth, Katja Eggermann, Christian Eggers

**Affiliations:** 1 Department of Neurology, Kepler University Hospital, Krankenhausstr. 9, 4020 Linz, Austria; 2 Department of Human Genetics, University Hospital RWTH Aachen, Pauwelsstraße 30, 52074 Aachen, Germany

**Keywords:** Motor neuron disease, Hereditary spastic paraplegia, PNPLA6, Novel variants, Novel phenotype, Cerebellar oculomotor dysfunction

## Abstract

**Objectives:**

The term hereditary spastic paraplegia comprises an ever-expanding array of neurological disorders with distinct aetiologies. Spastic paraplegia gene 39 is one of the many genetically defined types with features of other organs and neurological systems in addition to paraspasticity. We describe a large kindred with a novel clinical phenotype as, in addition to spastic paraplegia, affected subjects suffered from a prominent cerebellar oculomotor dysfunction with two hitherto undescribed mutations of *PNPLA6*.

**Methods:**

Three of five genetically tested family members of a large kindred were affected by spastic gait and a unique and prominent cerebellar oculomotor dysfunction. Further clinical, imaging, laboratory and videonystagmographic data were analyzed. Genetic analysis was done using next-generation sequencing.

**Results:**

The most salient clinical feature, in addition to paraspasticity, in three of five subjects was cerebellar oculomotor dysfunction with an upbeating nystagmus provoked by downward gaze. Genetic analysis revealed two hitherto unknown sequence variants in the *PNPLA6* gene, a splice-site variant c.1635 + 3G > T and a missense variant c.3401A > T, p.(Asp1134Val). In addition to cerebellar oculomotor dysfunction, compound-heterozygous siblings presented with paraspasticity and a moderate hypogonadotropic hypogonadism in the female. A paternal uncle being homozygous for the splice-site variant of *PNPLA6* presented with increased lower limb reflexes and an unstable gait. Treatment with 4-aminopyridine, a potassium channel blocker, lead to meaningful improvement of clinical symptoms.

**Conclusions:**

The unique and prominent cerebellar ocular motor disorder in our family broadens the spectrum of clinical phenotypes associated with variations in the PNLA6 gene. The finding of paraspasticity with cerebellar oculomotor dysfunction alongside inconspicuous brainstem imaging may raise suspicion of complex HSP with PNPLA6 mutations.

**Supplementary Information:**

The online version contains supplementary material available at 10.1007/s00415-022-11313-6.

## Introduction

Hereditary spastic paraplegias (HSPs), also known as Strümpell–Lorrain disease, are a heterogeneous group of neurodegenerative disorders. Their unifying clinical presentation is progressive spasticity and paraparesis, but there are many complex forms with other neurological and extra-neurological features [1]. Inheritance follows autosomal-dominant (AD), autosomal-recessive (AR) or X-linked patterns. The classification is based on the affected genes, hence the genetically based term spastic paraplegia gene (SPG) [2, 3]. So far, more than 80 causal genes or loci have been detected and resulted in a SPG classification [4, 5]. The common histopathological feature of HSPs is a length-dependent axonal degeneration of motor and sensory fibers within the corticospinal tract and the dorsal column [6, 7].

The phenotype of SPG39 corresponds to SPG20, an AR form of HSP with lower motor neuron involvement, and to organophosphorous compound-induced delayed neuropathy (OPIDN) with distal axonal degeneration [8–10]. SPG39 is caused by mutations in *PNPLA6*, a gene encoding neuropathy target esterase (NTE). Interestingly, mutations of *PNPLA6* have also been found to cause other rare hereditary neurodegenerative diseases with additional extra-neurologic features, such as Gordon–Holmes, Boucher–Neuhauser, Laurence–Moon and Oliver–McFarlane syndrome [11–13].

In 2008, Rainier et al. [8] reported two families in which affected subjects developed a childhood onset progressive spastic weakness of lower limbs and wasting of distal upper and lower limb muscles. In the consanguineous family, affected subjects carried a homozygous mutation in the *PNPLA6* gene c.3034A > G, p.(M1012V) that was shown to alter the catalytic domain of the encoded protein NTE. In the other, non-consanguineous family affected subjects were compound heterozygotes, where one allele had a missense mutation c.2669G > A, p.(R890H) and the other allele had an insertion mutation c.2946_2947insCAGC, p.(S982fs1019).

In 2014, Synofzik et al. [11] reported a family with HSP harboring the compound heterozygous variants c.787G > A, p.(Val263Ile) and c.2519G > A, p.(Gly840Glu) in the *PNPLA6* gene. Affected subjects presented with spasticity, hyperreflexia of lower extremities, positive Babinski’s sign and a mild motor neuropathy.

Yoon et al. [14] presented a sole patient with a pure spastic paraplegia phenotype characterized by motor delay and gait abnormalities with falls. This patient was heterozygous for the variant c.2944_2947dup, p.(Arg983ArgfsX86) of the *PNPLA6* gene.

We here describe a large kindred with two hitherto undescribed mutations in the *PNPLA6* gene. Their clinical phenotype was novel as, in addition to spastic paraplegia, they had a marked cerebellar oculomotor dysfunction.

### Case presentation

S1 (Subject 1), the index subject, was a 29-year-old male presenting with benign peripheral paroxysmal vertigo. Routine neurologic examination revealed marked gait disturbance for which the patient had never sought medical advice. He reported normal milestones during childhood and worked as a skilled specialist in manufacturing.

On examination he had grossly spastic gait with normal Romberg test and brisk muscle stretch reflexes with sustained ankle clonus. However, Babinski’s sign was negative. His muscles were neither weak nor atrophic. Oculomotor testing revealed massive saccadic pursuit movements. There was coarse nystagmus evoked by upward and horizontal gaze. On downward gaze, however, the direction of the nystagmus was upward (to his forehead) (Supplementary Table 1; Supplementary Video). There was no nystagmus in primary position. Rebound nystagmus could easily be elicited. HIT (head impulse test) according to Halmagyi showed normal vestibular ocular reflexes (VOR), but the suppression of the VOR by fixation was markedly impaired, indicating cerebellar dysfunction. Optokinetic nystagmus was reduced. Despite these prominent findings the patient had no subjective visual complaints. The remainder of the neurologic examination was normal. In particular there was no sensory, autonomic or cognitive dysfunction, no disorder of speech and fine finger movements, and no ataxia of gait or the upper limbs.

In S2 (subject 2), his 35-year-old sister, an office employee with good cognitive abilities, a marked gait disturbance had been present from early childhood with a very slowly progressive course. In terms of quality, her neurological findings were identical to subject S1; however, somewhat more pronounced. At the age of around 18 years, she developed secondary amenorrhea. Videonystagmography (VNG), done in S1 and S2, revealed a grossly reduced ability of fixation to suppress the VOR (Fig. 2). The father, S5 (subject 5), of S1 and S2 had no complaints. On examination he had sustained ankle clonus without Babinski’s sign. Gait and balance were normal and there was no oculomotor disturbance.

The same was true for the mother, S4 (subject 4), in whom unsustained ankle clonus was present.

S3 (subject 3), the 71-year-old paternal uncle of S1 and S2, became symptomatic only in his early 40s with gait disturbance and vertigo on head movement. On examination there was gaze evoked nystagmus to both sides and upwards but not downwards, saccadic pursuit movements, moderately impaired VOR and impairment of fixation-induced suppression of the VOR (corresponding with impaired reading on smooth sinusoid head movements).

Gait was clumsy but not spastic or atactic; Romberg test was normal. Muscle strength was normal without any atrophy, but knee and ankle jerks were markedly increased with sustained ankle clonus. Babinski’s sign was negative. There were no sensory findings.

The son of S3 had no complaints and was normal on thorough neurological examination.

In the extended family one female cousin in the paternal line of S1 and S2 had died from bulbar-onset amyotrophic lateral sclerosis at the age of 33, and another cousin had a well-founded diagnosis of multiple sclerosis. Genetic material was not available from these two subjects.

### Ethics approval

This study was approved by the local ethics committee of Johannes Kepler University Linz (#1105/2020).

### Paraclinical findings

Routine blood tests were done in S1, S2 and S3 and did not yield any systematic abnormalities. Hormone status was compatible with hypogonadotropic hypogonadism in S1 and S2.

In the CSF, taken from S1, routine parameters as well as neurofilament light, total tau and phosphorylated tau were unrevealing.

In S1 and S3, standard MRI (Siemens Avanto; 1,5 Tesla) of the brain and the entire spinal cord was normal without cerebellar or spinal cord atrophy. MRI or other radiological examinations were not available from the other family members.

Nerve conduction studies, done in S1 and S2, were conspicuous only for increased F-latencies in the peroneal and tibial nerves of only S1. Needle EMG was normal. In S2, magnetic evoked potentials showed increased central motor conduction times to the distal legs, while there were no responses in S1.

Ophthalmologic examination, done in S2, was normal including optical coherence tomography (OCT). Degree of relationship, genotype and clinical phenotype of subjects are given in Table 1 (more detailed information Supplementary Table 1).

**Table 1:** Clinical and molecular findings of subjects

Subject	Sex	Age	Relative to index subject	Mutation	Zygosity	Increased lower limb reflexes; sustained ankle clonus ^c^	Gait abnormality	Cerebellar oculomotor disturbance	Hypogonadotropic hypogonadism
S1	M^b^	29	Index subject	c.1635 + 3G > T c.3401A > T	Compound heterozygous	Yes (marked)	Yes	Yes (marked)	Yes
S2	F^a^	35	Sister	c.1635 + 3G > T c.3401A > T	Compound heterozygous	Yes (marked)	Yes	Yes (marked)	Yes
S3	M	71	Paternal uncle	c.1635 + 3G > T	Homozygous	Yes (marked)	Yes	Yes (marked)	None
S4	F	55	Mother	c.3401A > T	Heterozygous	Yes (marked)	None	None	None
S5	M	58	Father	c.1635 + 3G > T	Homozygous	Yes (marked)	None	None	None

^a^Female

^b^Male

^c^Tendon reflexes were graded by a reference scale from the National Institute of Neurological Disorders and Stroke (NINDS)

## Materials and methods

The study was approved by the local ethics committee, and subjects provided written informed consent (Declaration of Helsinki).

For videonystagmography (VNG), the Autronic VOG system (Autronic GmbH, Hamburg, Germany) was used, and measurements were carried out by the same examiner. After calibrating the system, tests were conducted before and 1 h after the intake of 10 mg 4-aminopyridine in Subject 1.

Capturing genes under investigation, an Illumina Enrichment Kit (Panel-ID 109980) was used. The generated library was sequenced on a MiSeq sequencer (Illumina). The generation of the analyzed "bam" or “FastQ” files was carried out with the MiSeqReporter Software (v.2.6.2, Illumina, reference genome: hg19). The generated raw data were analyzed using the SeqNext module of the SeqPatient software (JSI, v.4.3.0-B503). Variants with a frequency of the rarer allele of > 1% in relevant databases (EXAC, dbSNP, 1000G), synonymous variants and variants that are not within the coding region, outside of the canonical splice sites, as well as variants which are not bi-directionally covered were not considered. The following genes were analyzed using the Illumina Enrichment Kit: *ATL1* (SPG3)*, SPAST* (SPG4), *CYP7B1* (SPG5), *PGN* (SPG7), *KIF5A* (SPG10), *SPG11* (SPG11), *ZFYVE26* (SPG15), *REEP1* (SPG31), *ACP33*, *AMPD2*, *AP4B1*, *AP4E1*, *AP4M1*, *AP4S1*, *AP5Z1*, *ARL6IP1*, *ARSI*, *B4GALNT1*, *C12ORF65*, *C19ORF12*, *CAPN1*, *CYP2U1*, *DDHD1*, *DDHD2*, *ENTPD1*, *ERLIN1*, *ERLIN2*, *FA2H*, *GAD1*, *GBA2*, *GJC2*, *HSPD1*, *KCNA2*, *KIAA0196*, *KIAA1840*, *KIF1C*, *KLC2*, *L1CAM*, *NIPA1*, *NT5C2*, *PGAP1*, *PLP1*, *PNPLA6*, *RAB3GAP2*, *REEP2*, *RTN2*, *SLC16A2*, *SLC33A1*, *SPG20*, *USP8*, *VPS37A*, *WDR48*, *ZFR*, *ZFYVE27*; Coverage was > 30X for approximately 99% of the regions of interest.

For segregation analyses of the variants, the relevant sequences of the *PNPLA6* gene were PCR-amplified and subsequently sequenced using standard Sanger sequencing protocols. To exclude copy number variants in *REEP1*, *SPG7*, *SPAST* and *ATL1*, a semi-quantitative MPLA (Multiplex Ligation Dependent Probe Amplification) method (Kits no. P231-B2 and P165-C2, MRC-Holland) was used.

For bioinformatic assessment of pathogenicity Alamut v.2.15.0 and PolyPhen-2 were used. It must be considered that these programs only provide information on possible pathogenicity. The pathogenicity assessment of genomic variants is essentially based on the consensus recommendations of the ACMG (American College of Medical Genetics and Genomics) [15].

### Data sharing

Data are available upon request to the corresponding author.

## Results

In S1 and S2 next generation sequencing (NGS) identified two variants in the PNPLA6 gene (OMIM: 603197; NM_001166111): a missense mutation c.3401A > T, p.(Asp1134Val) and a splice-site variation c.1635 + 3G > T possibly influencing the donor splice-site of intron 15. To date, neither of these sequence alterations have been described in the literature or in databases. The missense variant c.3401A > T in exon 29 is non-synonymous, i.e., it leads to the change of the amino acid Asp (aspartate) to Val (valine), and it affects a highly conserved part of the catalytic centre of the NTE.

Bioinformatics and in silico assessments by online tools showed a high likelihood of a deleterious effect of this mutation on the structure and function of its protein.

The substitution in intron 15 might lead to aberrant splicing according to splice-site prediction tools. No further pathogenic or possibly pathogenic variants were detected. MLPA did not show any deletions or duplications of single exons of *REEP1*, *SPG7*, *SPAST* and *ATL1*.

**Fig. 1:**
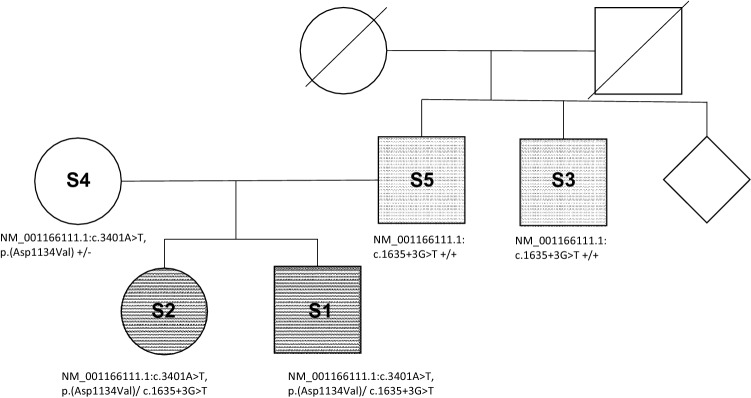
Partial pedigree of *PNPLA6* family with novel genotype and phenotype. Pedigree of the *PNPLA6* family; Affected subjects are shown as filled symbols. Compound-heterozygous subjects (S1, S2) presented with the novel genotype and phenotype consisting of a marked cerebellar oculomotor dysfunction, lower limb spasticity and a hypogonadotropic hypogonadism are shown in black. Grey symbols present subjects S3 and S5 suffering from a milder phenotype

Sanger sequencing confirmed heterozygosity for each of the detected variants in patients S2 and S1 (Supplementary Fig. 1a, b). Segregation analysis revealed compound-heterozygosity and indicated AR inheritance. S4 showed heterozygosity for the missense variant c.3401A > T, p.(Asp1134Val) assuming her as heterozygous carrier of the possibly disease-causing sequence alteration in *PNPLA6* gene, whereas S5 and S3 were homozygous carriers of the splice-site variant c.1635 + 3G > T. A partial family pedigree is given in Fig. 1.

Taken together, the clinical symptoms of siblings S1 and S2, who shared the same variations in *PNPLA6* gene (c.1635 + 3G > T/c.3401A > T, p.Asp1134Val) as a compound heterozygosity, consisted of lower limb spastic paraparesis and a marked cerebellar oculomotor disorder accompanied by hypogonadotropic hypogonadism. In contrast, their father, a homozygous carrier of the *PNPLA6* variant c.1635 + 3G > T, was free of complaints but had sustained ankle clonus. His older brother S3 with the same homozygous mutation had gait disturbance with increased stretch reflexes and an oculomotor dysfunction of the cerebellar type. The mother S4, a heterozygous carrier of c.3401A > T, p.(Asp1134Val) was free of complaints but she had a somewhat brisk ankle reflex without sustained clonus. Oculomotor function in both parents was normal.

**Fig. 2:**
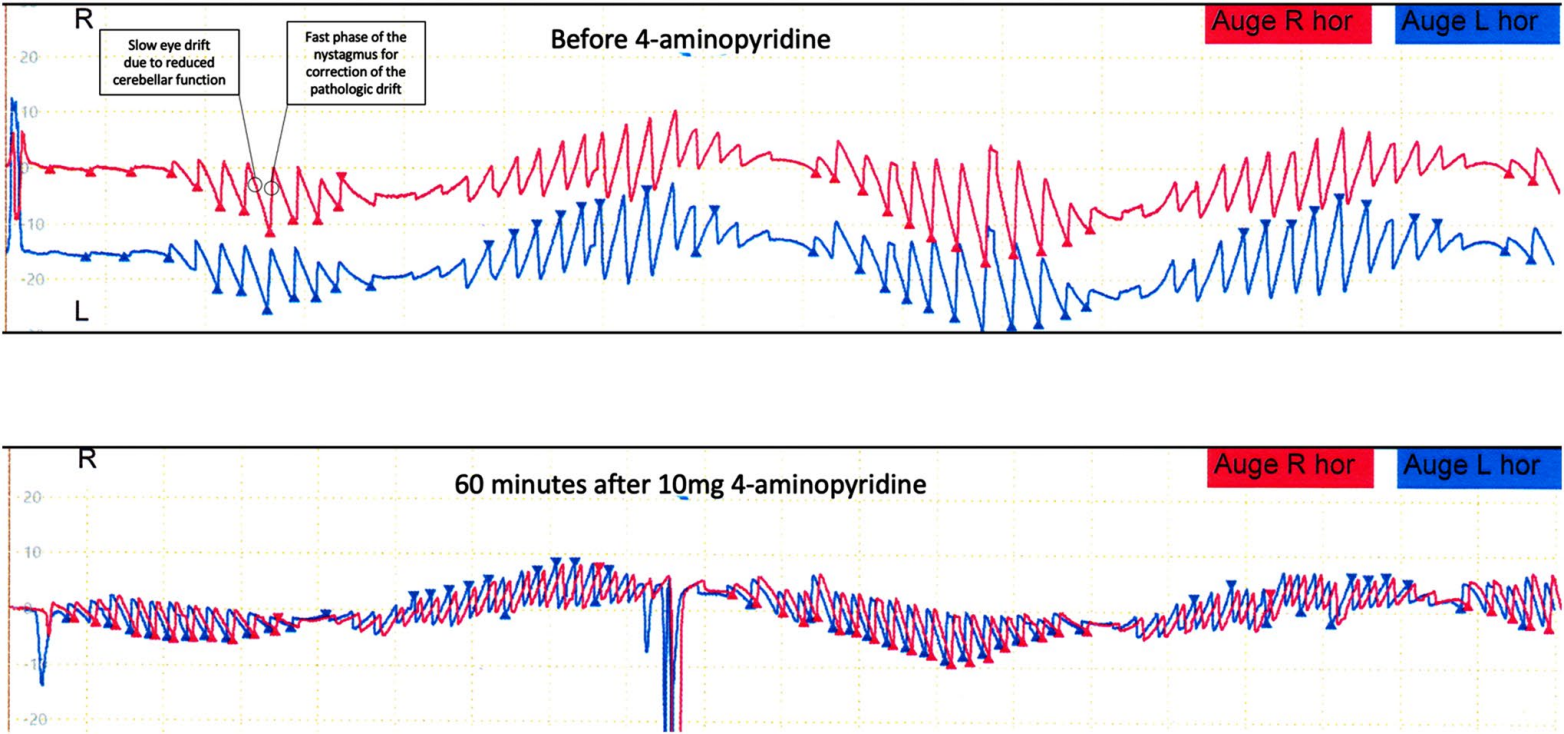
Suppression by fixation of the VOR before and after 4-aminopyridine in VNG. Suppression by fixation of the VOR, before and after 4-aminopyridine. The seated subject is repeatedly turned around his vertical axis with sinusoidal movements while fixating a visual target. The visual target is moving in parallel with his body and head so that, to continuously fixate the target, the subject needs to keep his eyes in the primary position. Without 4-aminopyridine a high-amplitude drift with corresponding nystagmus is seen in both (horizontal) directions. 60 min after 10 mg 4-aminopyridine the amplitude of drift and nystagmus is significantly reduced. The angular velocity and amplitude of rotation were kept the same in the two examinations

4-aminopyridine 10 mg t.i.d (ter in die) was given to subjects S1, S2 and S3 who requested symptomatic treatment. All reported meaningful improvement in dexterity and steadiness of gait. In S1, VNG performed when the patient was off the drug and 60 min after the intake of 10 mg 4-aminopyridine (4-AP), revealed improvement of his cerebellar system to suppress the VOR. With 4-AP, turning the head while fixating a target moving in parallel with his head elicited a nystagmus that was lower in amplitude (Fig. 2).

## Discussion

Among the more than 80 different genetic causes of HSP, SPG39, which is caused by alterations in the gene *PNPLA6*, is likely to be one of the rarest forms. While *PNPLA6* has been implicated in a variety of disorders, among which the leading manifestations are often extra-neural, spastic paraplegia as the leading or even sole manifestation has been described in only three case reports. *PNPLA6* causes different neurodegenerative motor diseases with a wide spectrum of symptoms. Rainier et al. [8] postulated that complex HSP, i.e., clinical features in addition to spasticity, such as distal muscle wasting, is the most common phenotype of *PNPLA6* mutations. However, Synofzik et al. [11] showed that besides complex HSP which is “the peak of the iceberg”, *PNPLA6* can cause a spectrum of neurodegenerative disorders with four main clinical features: ataxia, chorioretinal dystrophy, spasticity and hypogonadism. So far, cerebellar oculomotor dysfunction has not been associated with *PNPLA6* mutations.

The family reported here has a novel clinical phenotype with novel compound heterozygous variants in *PNPLA6*. Apart from lower limb spasticity with spastic gait, the most salient feature was marked oculomotor dysfunction comprising saccadic pursuit movements, rebound nystagmus and impaired suppression of the VOR by fixation.

The most prominent finding was a peculiar gaze evoked nystagmus. While the fast phase of the nystagmus was beating into the direction of horizontal and upward gaze, this was reverted on downward gaze. Here, the direction of the fast phase of the nystagmus was upwards (to the forehead). While these findings may be classified as cerebellar dysfunction, other cerebellar features, such as gait ataxia, ataxia of upper limbs or dysarthric speech were conspicuously absent. As the sole non-neurologic feature, patient S1 and S2 had low concentrations of blood gonadotropic hormones, indicating mild hypogonadotropic hypogonadism. Taken together, the organ systems affected to the extent of becoming clinically apparent were the cortical motor neurons, cerebellar neurons or connections and, to a much lesser extent, the reproductive endocrine system. Hufnagel et al. [13] found individual tissues to express different levels of NTE activity. Thus, in our patients, NTE activity was likely most prominently reduced in cortical motor and cerebellar neurons.

Pathogenic variants of *PNPLA6* hitherto described all affect genetic sequences that code for the catalytic domain of NTE and that are highly conserved across species. There are a variety of *PNPLA6* phenotypes with specific combinations of clinical features, such as spastic ataxia (spasticity & ataxia), Gordon Holmes syndrome (spasticity, primarily gait ataxia & hypogonadism), Boucher–Neuhäuser syndrome (ataxia, hypogonadism & chorioretinal dystrophy) and pure HSP (spasticity) [11]. With an upper motor neuron disorder, isolated oculomotor cerebellar dysfunction and a limited degree of hypogonadism we add a novel phenotype that is likely to be caused by the detected variants of *PNPLA6*.

In our subjects S1 and S2 with compound heterozygosity the onset was earlier and the clinical symptoms more severe than in their father S5 and uncle S3 who both were homozygous for the splice-site variant c.1635 + 3G > T. This might suggest a more deleterious impact of the missense variant c.3401A > T than of the splice-site variant c.1635 + 3G > T. Although S3 and S5 carried the same novel splice-site variant the older brother S3, with cerebellar dysfunction and spasticity, was clinically more affected than his younger brother S5 who only had brisk reflexes. This finding corresponds to previous findings of Synofzik et al. [11] who found weak genotype–phenotype correlations. Although S3 was 13 years older than S5 he had become symptomatic already at the age of 40, when he was 18 years younger than S5 who still had neither a cerebellar nor gait disturbance. A further possible explanation for the discrepancy between genotype and phenotype in S3 and S5 might be epigenetic factors influencing the splicing process of *PNPLA6* gene. Our findings mirror those of previous studies, where the clinical phenotype was found to be neither dependent on the location within the gene nor the type of the pathogenic variant [11, 13, 16]. Hence, a genotype–phenotype correlation between our novel *PNPLA6* variants and clinical phenotypes cannot be postulated.

Subjects with NTE alterations frequently suffer from progressive upper more than lower motor neuron disease. This has led to the assumption that NTE plays a major role in the integrity of motor axons [8]. Amyotrophic lateral sclerosis (ALS) is a similar progressive degenerative motor neuron disorder. Rainier et al. [8] considered variations of the NTE to contribute to the pathogenesis of ALS. Interestingly, a cousin of subject S1 and S2 from the paternal line died of bulbar-onset ALS at the age of 33. This raises speculations that her ALS was based on a *PNPLA6* mutation; however, she did not undergo genetic testing.

4-Aminopyridine, a potassium channel blocker that is being used in downbeat nystagmus syndrome, a similar disorder of cerebellar nuclei and connections, proved to be clinically beneficial in our patients.

To summarize, our observations expand the already wide spectrum of clinical phenotypes reported with alterations in the *PNPLA6* gene. The hitherto undescribed (compound heterozygous) *PNPLA6* variants were accompanied by a novel clinical phenotype comprising prominent dysfunction of cerebellar oculomotor structures and the upper motor neuron system as well as some degree of hypogonadotropic hypogonadism.

## Supplementary Information

Below is the link to the electronic supplementary material.

Supplementary file1 (DOCX 78 KB)

Supplementary file2 (MP4 54382 KB)

## Abbreviations

AD: Autosomal-dominant
AR: Autosomal-recessive
HIT: Head impulse test
HSP: Hereditary spastic paraplegia
MLPA: Multiplex ligation dependent probe amplification
NTE: Neuropathy target esterase
OCT: Optical coherence tomography
OPIDN: Organophosphorous compound-induced delayed neuropathy
SPG: Spastic paraplegia gene
SPG39: Spastic paraplegia-39
VNG: Videonystagmography
VOR: Vestibular-ocular reflex
