# Supplementary material for: Novel phenotype with prominent cerebellar oculomotor dysfunction in spastic paraplegia type 39
Source: J Neurol. 2022 Aug 10;269(12):6476–82. doi: 10.1007/s00415-022-11313-6 (PMC9618546; doi:10.1007/s00415-022-11313-6)
Supplement: Supplementary file 1 — Supplementary file1 (DOCX 78 KB) [file 415_2022_11313_MOESM1_ESM.docx]

**
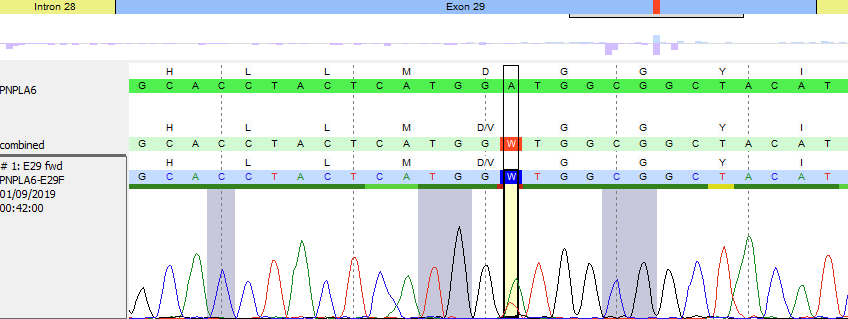

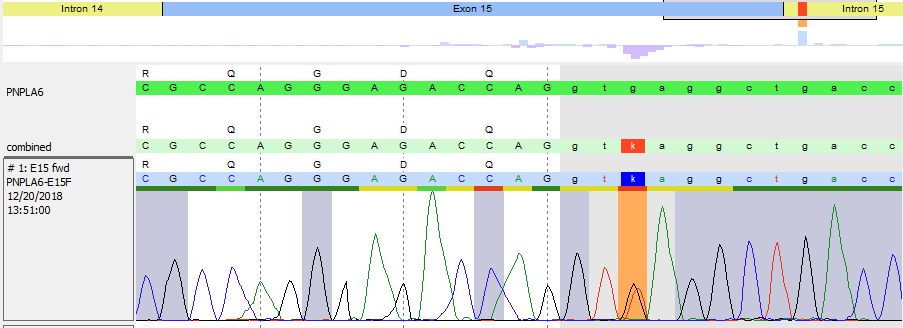
**

**Supplementary Figure 1b. Results of Sanger sequencing in the index subject S2 of *PNPLA6* missense variant**

**Supplementary Figure 1a. Results of Sanger sequencing in the index subject S2 of PNPLA6 splice-site variant**

| **Supplementary Table 1. More detailed clinical and molecular findings of our subjects compared to previously published case reports** | | | | | | | | |  |
| --- | --- | --- | --- | --- | --- | --- | --- | --- | --- |
|  | **Present family** | | | | | **Synofzik et al. (2014)** | **Rainier et al. (2008)** | | **Yoon et al. (2013)** |
|  | **S1** | **S2** | **S3** | **S4** | **S5** | **IHG 26041** | **Nonconsanguineous family** | **Consanguineous family** | **Subject ID 16** |
| **Origin** | A | A | A | A | A | G | USA | USA/Ashkenazi | n.a. |
| **Gender** | m | f | m | f | m | f | f/f/m | f/m | m |
| **Phenotypic syndrome** | HSP | HSP | HSP | H | H | HSP | HSP | HSP | HSP |
| **Age at examination** ^a^ | 29 | 35 | 71 | 55 | 58 | 54 | n.a. | n.a. | 8 |
| **Age at onset** ^a^ | 20 | 20 | 40 | n.a. | n.a. | 20 |  |  | 6 |
| **First symptom** | LL Spasticity | LL Spasticity | Unstable gait | n.a. | n.a. | Spasticity | LL spastic weakness | LL spastic weakness | Gait abnormality |
| **Saccadic horizontal eye movements** | + | + | + | - | - | n.a. | - | - | - |
| **Gaze-evoked nystagmus** | + | + | + | - | - | - | - | - | - |
| **Downward gaze provoked upbeat nystagmus** | + | + | - | - | - | - | - | - | - |
| **Rebound nystagmus** | + | + | + | - | - | - | - | - | - |
| **Optokinetic nystagmus** | + | + | + | - | - | - | - | - | - |
| **Pathological fixation suppression of VOR** | + | + | + | - | - | - | - | - | - |
| **Deep tendon reflexes** ^b^ | ↑/↑ | ↑/↑ | ↑/↑ | ↑/↑ | ↑/↑ | ↑/↑ | n.a. | n.a. | n.a. |
| **Sustained ankle clonus** | + | + | + | + | + | n.a. | n.a. | n.a. | n.a. |
| **Distal muscle wasting** | - | + | - | - | - | - | + | + | - |
| **Spasticity LL/ Extensor plantar response** | +/- | +/- | +/- | -/- | -/- | +/+ | +/n.a. | +/n.a. | n.a./n.a. |
| **Sensory system** | n | n | n | n | n | n.a. | n.a. | n.a. | n |
| **Cognitive impairment** ^c^ | n | n | n | n | n | n | n | n | n |
| **Gait ataxia** | - | - | - | - | - | - | - | - | - |
| **Hypogonadotropic hypogonadism** | + | + | - | - | - | - | n.a. | n.a. | - |
| **Chorioretinal dystrophy** | - | - | - | - | - | - | n.a. | n.a. | - |
| **Hypercholesterinemia** | + | n.a. | - | n.a. | n.a. | n.a. | n.a. | n.a. | n.a. |
| **Liver parameters** | ↑ | n.a. | n | n.a. | n.a. | n.a. | n.a. | n.a. | n.a. |
| **Impaired Blood-brain barrier** | + | n.a. | - | n.a. | n.a. | n.a. | n.a. | n.a. | n.a. |
| **Neurofilaments CSF** | n | n.a. | n.a. | n.a. | n.a. | n.a. | n.a. | n.a. | n.a. |
| **F-Wave LL** | ↑ | n | n.a. | n.a. | n.a. | n.a. | n.a. | n.a. | n.a. |
| **Motor evoked potentials** | ↑↑ | ↑ | n.a. | n.a. | n.a. | n.a. | n.a. | n.a. | n.a. |
| **Brain MRI** | n | n.a. | n | n.a. | n.a. | n | Spinal cord atrophy | Spinal cord atrophy | n.a. |
| **Mutations** | c.3401A>T; c.1635+3G>T  (compound-heterozygous) | c.3401A>T; c.1635+3G>T (compound-heterozygous) | c.1635+3G>T  (homozygous) | c.3401A>T  (heterozygous) | c.1635+3G>T  (homozygous) | c.2519G>A;c.787G>A  (compound heterozygous) | c.2669G>A;  c.2946_2947insCAGC  (compound-heterozygous) | c.3034G>A  (homozygous) | c.2944_2947dup  (heterozygous) |

^a^ in years

^b^ Tendon reflexes were graded by a reference scale from the National Institute of Neurological Disorders and Stroke (NINDS).

^c^ Cognitive impairment assessed by clinical presentation

A = Austria; G = Germany; USA = United States of America; m = male; f = female; H = Healthy; n.a. = not available; LL = lower limbs; n = normal; + = positive; - = negative; ↑ = increased; ↑↑ = highly increased;
